# Osteolipoma: An Extremely Rare Hard Palate Tumor

**DOI:** 10.7759/cureus.8146

**Published:** 2020-05-15

**Authors:** Sonam Sharma, Vikas Dhillon

**Affiliations:** 1 Pathology, Kalpana Chawla Government Medical College, Karnal, IND; 2 Otorhinolaryngology, Kalpana Chawla Government Medical College, Karnal, IND

**Keywords:** lipoma, osseous, oral cavity, hard palate

## Abstract

Lipoma and its variants rarely involve the oral cavity. Osteolipoma of the hard palate is extremely uncommon with only a few cases reported worldwide. It is important to recognize and give comprehensive diagnosis of this entity, so as to prevent unwarranted medical interventions. Here, in this report, we describe a rare case of osteolipoma arising in the hard palate of a 35-year-old male and the diagnostic conundrum associated with it. The approach to such a case, differential diagnosis, and review of the literature are also presented.

## Introduction

Lipoma is the most common benign soft tissue mesenchymal neoplasm in adults. It has a tendency to occur at almost any anatomical location of the body where fat normally exists, therefore, it is also known as a ubiquitous or universal tumor [1]. According to the existing literature, lipoma seldom occurs in the oral cavity, where it comprises of only 1%-4% of all the benign oral lesions and primarily affects the buccal mucosa, tongue, floor of the mouth, lips, and gingiva [2]. The incidence of involvement of the hard palate by lipoma is very low as it has a very little fat content [3]. Osteolipoma, a lipoma exhibiting bone formation, is a very rare histological variant of lipoma, accounting for less than 1% of the cases [4]. This uncommon neoplasm is frequently found at the extraoral sites and till date, only a handful of intraoral osteolipoma cases have been documented. The paucity of knowledge about intraoral osteolipoma along with the lack of understanding about its genesis at unusual locations prompted us to present such an enigmatic entity by describing an additional case of osteolipoma uniquely located in the hard palate of an adult male.

## Case presentation

**Figure 1:**
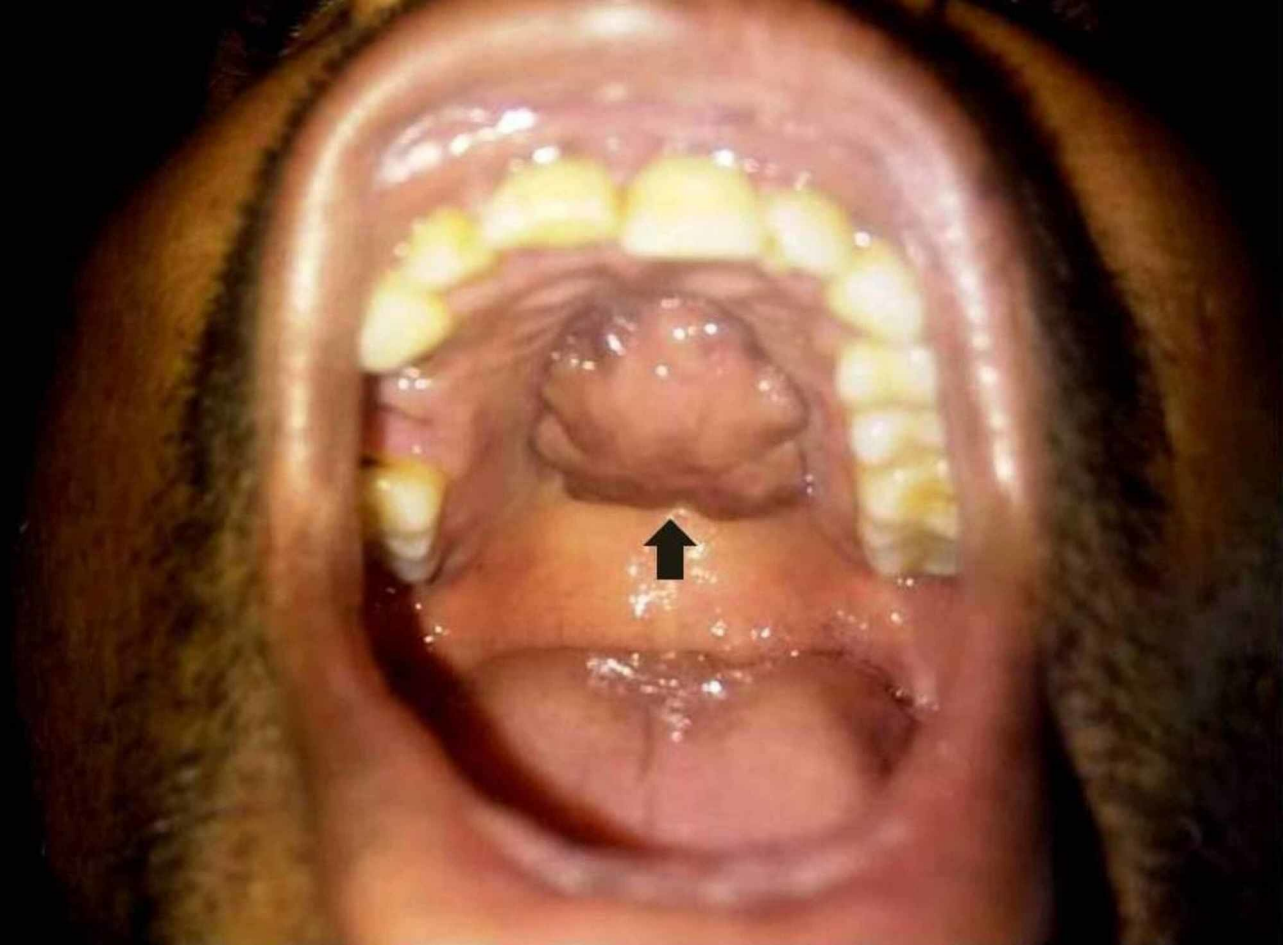
Hard palate growth (black arrow) on intraoral clinical inspection.

A 35-year-old male presented to the otorhinolaryngology outpatient department with the chief complaint of a painless palatal mass of eight-year duration. His history of present illness revealed that initially a pea-sized swelling was noticed by him inside the mouth on the hard palate. However, it gradually grew over the last four years to attain the present size. There was no history of local trauma, bleeding, pain, sensory changes, disturbance of salivation, any other oral lesions, fever, loss of appetite, or weight loss. The past dental, medical, and personal history were unremarkable. The family history was noncontributory. On general physical examination, he was of moderate built and all his vitals were within the normal limits. There was no regional lymphadenopathy. No abnormality was detected on his systemic examination. Extraoral examination was unremarkable. Opening of the mouth was adequate. Intraoral examination revealed the oral hygiene to be poor. A round to oval mass with irregular margins measuring 4 cm x 2.5 cm in size was seen on the hard palate (Figure 1).

**Figure 2:**
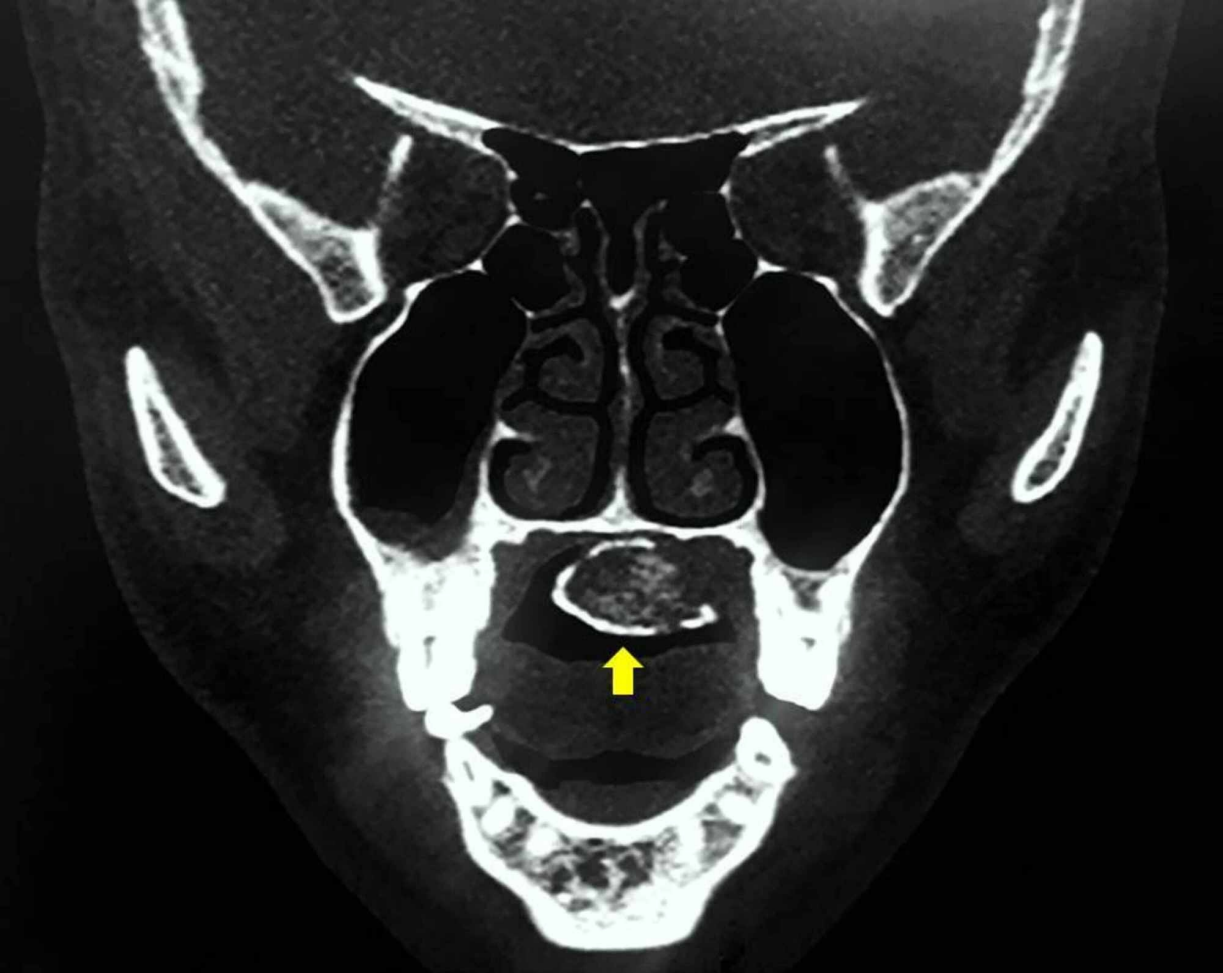
Coronal non-contrast CT scan of the face showing a well-circumscribed, fat-containing calcified hard palate lesion (yellow arrow).

On palpation, it was well defined, firm to hard in consistency, nontender, nonfluctuant, nonreducible, nonpulsatile, and immobile. The color of the overlying mucosa was same as that of the adjacent mucosa. On the basis of history and clinical findings a provisional diagnosis of torus palatinus was made. CT scan of the face revealed a well-defined lobulated soft tissue lesion with a peripheral rim of calcification along the inferior aspect of the hard palate. There was no evidence of invasion into the adjacent structures (Figure 2).

**Figure 3:**
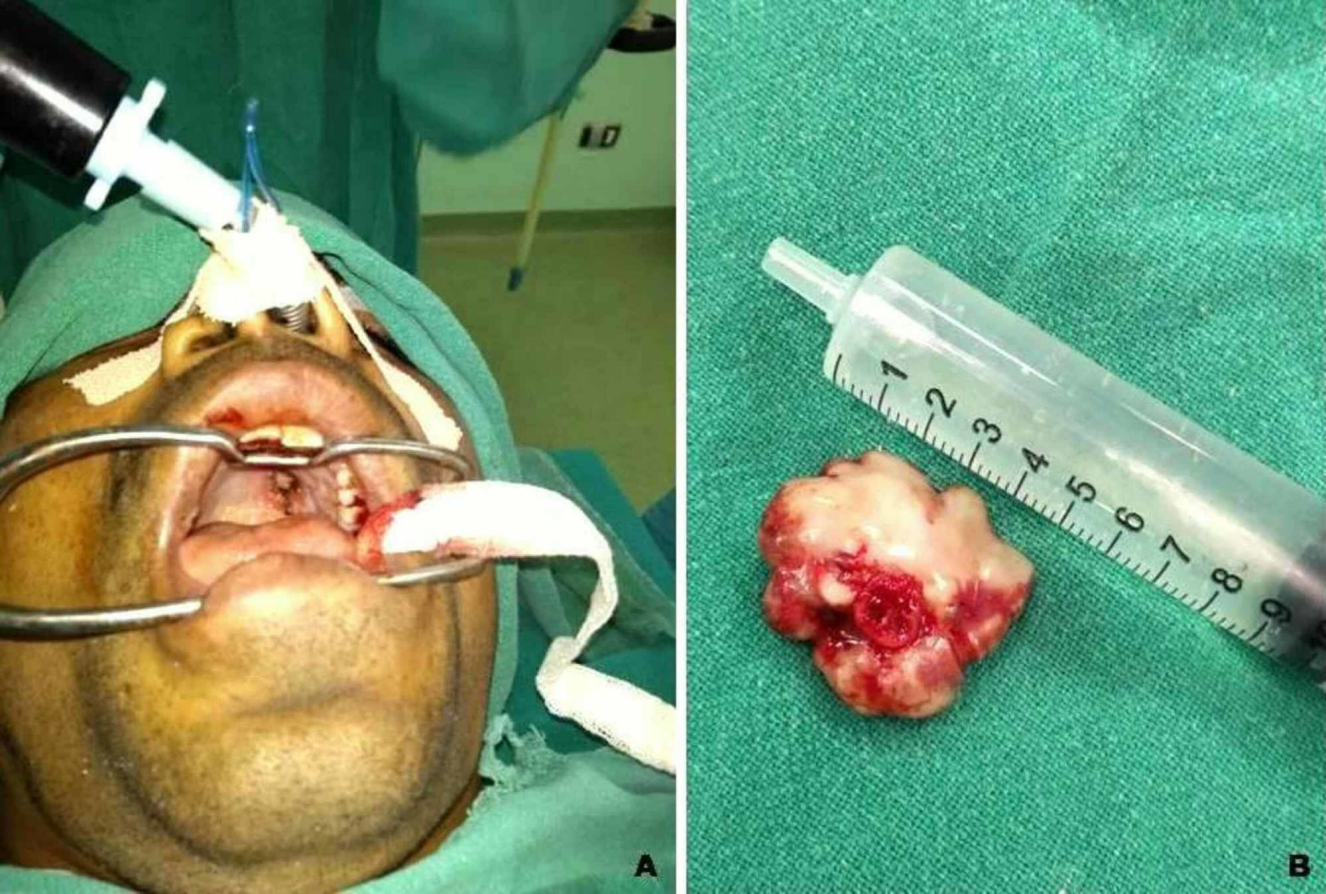
(A) Intraoperative view. (B) Resected hard palate mass.

Based on these radiological features, the possibility of a calcified hamartoma or an osseous choristoma was kept. Simultaneously, after his routine laboratory investigations to arrive at a conclusive diagnosis, fine needle aspiration cytology (FNAC) was performed. However, owing to the hardness of the mass, FNAC attempts failed to yield any material for establishing the diagnosis. The lesion was excised under general anesthesia using nasal intubation and the specimen was sent for histopathological examination (Figure 3).

**Figure 4:**
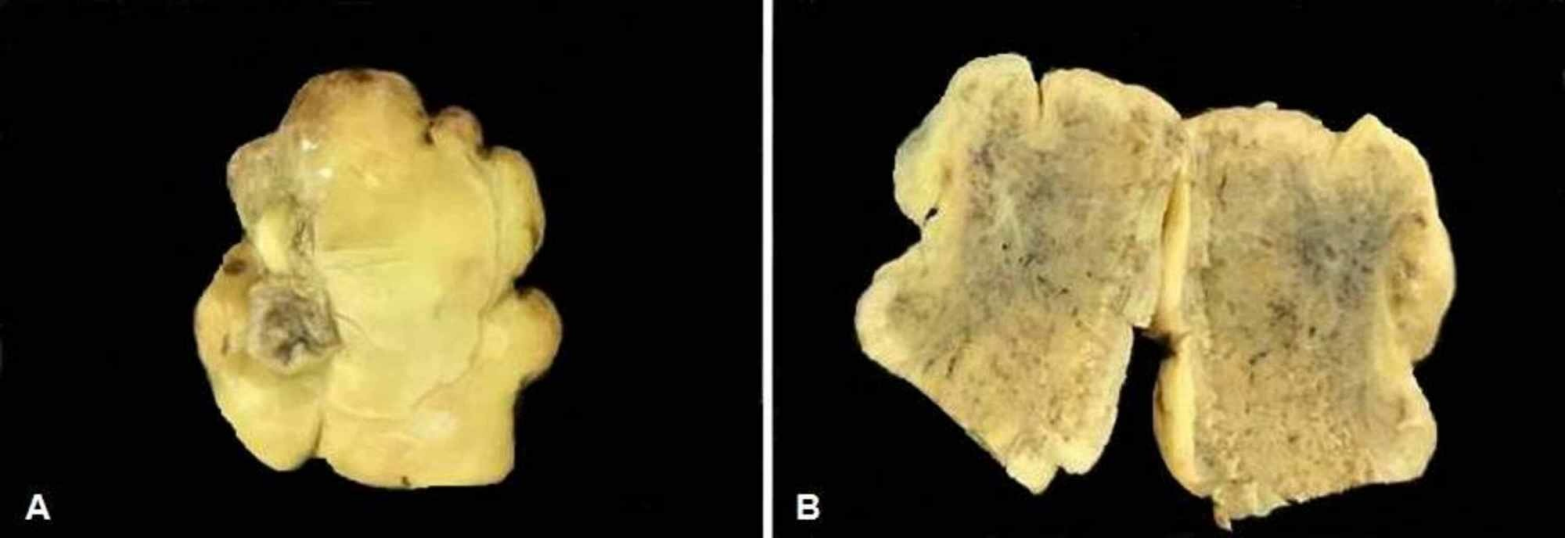
(A) Macroscopic decalcified specimen. (B) Yellow-gray hard cut surface.

Grossly, the mass was well demarcated, yellowish, hard in consistency, and measured 4 cm x 2.7 cm x 0.8 cm in size. The specimen was decalcified. It was gritty on cut and the sections were yellowish-gray (Figure 4).

**Figure 5:**
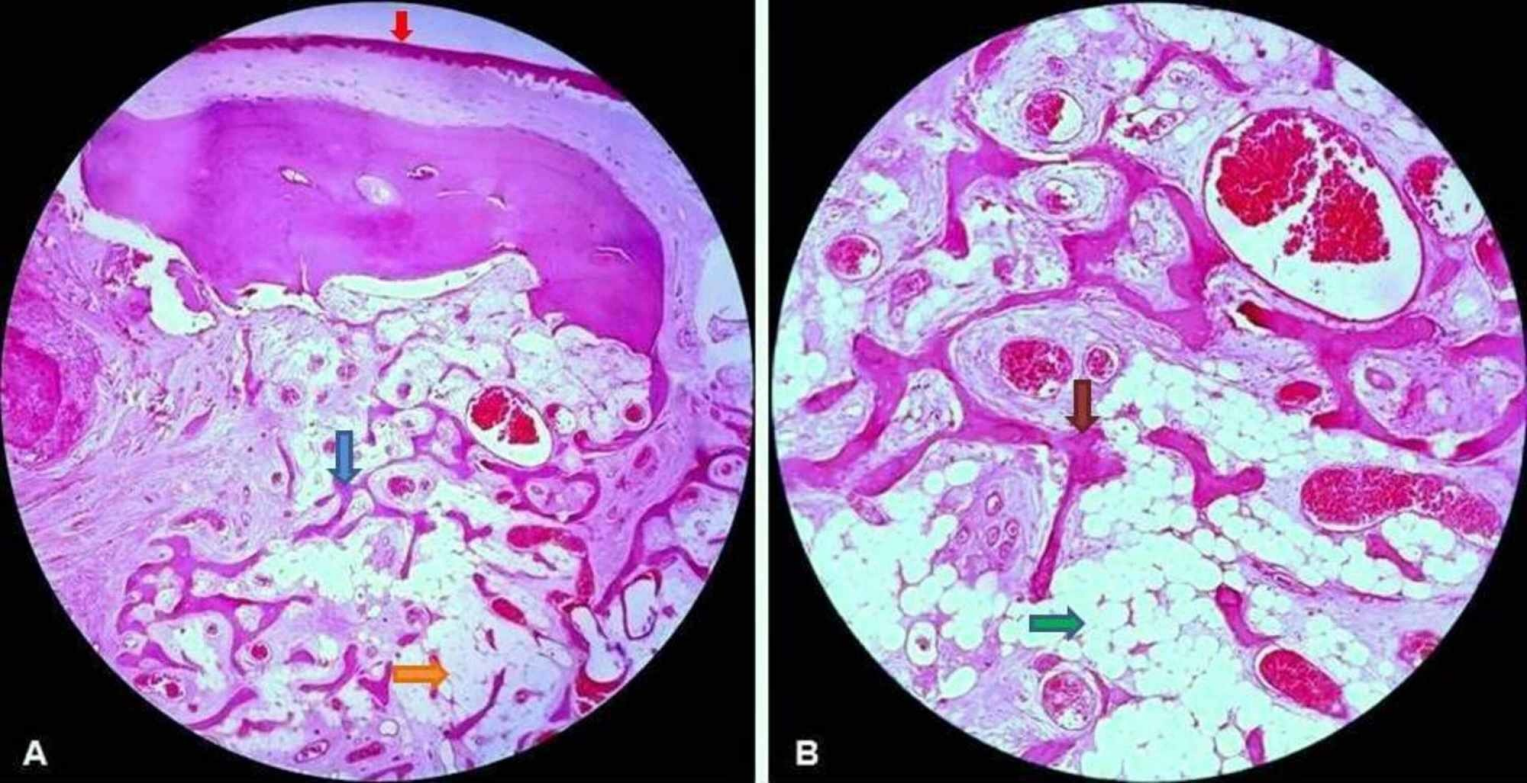
(A) Photomicrograph showing epithelial lining of hard palate (red arrow) with the underlying connective tissue exhibiting randomly distributed irregular trabeculae of immature bone (blue arrow) among benign looking adipocytes (orange arrow) (H and E, x100). (B) Higher magnification showing bony trabeculae (brown arrow) surrounded by mature adipocytes (green arrow) (H and E, x200).

Microscopic examination exhibited a tissue lined by keratinized stratified squamous epithelium. The subepithelium showed abundant mature adipose tissue comprising uniform adipocytes which were separated by thin connective tissue septa at places. Also, scattered among them were the variably sized and shaped normal bony trabeculae with features of osteoblastic rimming. No nuclear atypia, cell pleomorphism, mitosis, necrosis, or foci of hematopoietic cells were seen (Figure 5).

On the basis of all these histopathological features, a final diagnosis of hard palate osteolipoma was established. The postoperative period of the patient was uneventful and no recurrence of the lesion was detected even after three years of follow-up.

## Discussion

Intraoral lipoma is quite an uncommon neoplasm, accounting for 2.2% of all the lipomas and has a prevalence rate of 0.0002%. Roux in the year 1848, first described it in a review of alveolar masses, in which he referred it as a “yellow epulis” [5]. It is mainly encountered in males above the age of 40 years as a slow growing asymptomatic mass which can occur anywhere, the buccal mucosa being the most common site [6]. The occurrence of lipoma in the hard palate is extremely rare [7]. Pathogenetically, the development of lipoma has been linked with hereditary conditions, trauma as well as obesity and generally, it is composed of mature white adipocytes [1, 8]. Nevertheless, this tumor can occasionally show changes depending upon the predominance of other mesenchymal elements [9]. One such extremely uncommon variant of it is osteolipoma, which has been described as a lipoma containing osseous elements. This rare tumor can occur in the head and neck region, upper and lower extremities, sternoclavicular region, and in the subcutaneous soft tissue [10]. The exact pathogenesis of osteolipoma still remains unclear. However, two important theories have been proposed by researchers which can result in its development. These include multidirectional differentiation of multipotent mesenchymal cells and metaplasia of fibrous elements into bone tissue, secondary to the existing “classic” lipoma in response to various external factors such as mechanical stress, repeated trauma, or ischemia. Nevertheless, aside from these hypothesis, it has also been suggested that osteolipoma can arise due to the factors released by monocytes in the circulation that induce the transformation of fibroblasts into osteoblasts, or perhaps due to an inadequate nutritional supply in the center of a large lipoma [6, 11].

Intraoral osteolipoma, as seen in the present case study, is exceedingly rare. On further exploring the existing literature on intraoral osteolipoma, certain clinicopathological features of this condition draw special attention, which in turn have direct diagnostic and therapeutic implications. Clinically, any age group and gender can be involved. However, it is usually seen in adult males over 30 years of age. Various anatomical sites, regardless of any bone proximity can be involved by this tumor. According to the pertinent world literature till date, 15 cases of intraoral osteolipoma have been documented so far, with eight of them being reported from buccal mucosa, three from hard palate, two from floor of the mouth, one from lateral border of the tongue, and one from retromolar trigone [6,12]. To the best of our knowledge, our case is the fourth case to be reported from the hard palate.

The major presenting symptom is a painless, gradually growing, soft or hard, round or discoid, mobile or fixed, generally well-defined mass, the size of which can be variable and may range from 0.8 to 9 cm. The tumor usually remains undetected by the patient for years as there is usually a long interval between occurrence of the lesion and the time of presentation of the patient to the clinician [7,13]. This clinical presentation is consistent with the present case, which occurred in a 35-year-old male who reported that the lesion was a painless, hard, irregular, fixed growth, which had been present in the hard palate for a period of eight years. This asymptomatic nature of this tumor along with its inherent potential to occur at different intraoral locations makes the tumor diagnosis a challenge. A wide variety of benign and malignant lesions, have been included in its differential diagnosis such as oral osseous choristoma (soft tissue osteoma), cartilaginous choristoma, chondrolipoma, pleomorphic adenoma with ossification, and other salivary gland or connective tissue tumors with dystrophic calcification, osteosarcoma, synovial sarcoma, osteosarcoma, chondrosarcoma, teratoma, post-traumatic chondrification, exostosis, peripheral giant cell granuloma, fibrous hyperplasia, neurofibroma, intraosseous cysts, tumor calcinosis, central hemangioma, and myositis ossificans [2, 6-7]. Therefore, a high index of suspicion and collaboration of both the radiological and pathological investigations play an integral role in its diagnosis. Diagnostic techniques such as CT scan and MRI are able to examine and quantify the lesions internal components such as fat, soft tissue, and calcifications. CT scan is preferable for observing ossification, whereas MRI is helpful for observing soft tissue such as fat. On CT scan, this tumor appears as a well-defined mass containing areas of both low-density fatty tissue and high-density calcification, with thin septa, while the MRI shows a well-circumscribed tumor with high-signal intensity on T1-weighted imaging. Post-contrast T1-weighted imaging shows coarse calcifications with no significant nodular enhancement. On fat-suppressed T2-weighted images, the mass displays high-signal foci with uniform suppression of the contained fatty tissue. Overall, these radiological characteristics are important in distinguishing this tumor from more aggressive fat-containing lesions such as liposarcoma [11]. The role of FNAC in assessing intraoral lesions is still debatable and has variable outcomes [14-17]. With respect to intraoral osteolipoma, there is limited utility of cytology in diagnosing it due to its hardness, however, still it can provide a clue that the lesion that we are dealing can be of osseous origin. Therefore, histological examination is considered the best approach to reach a definitive diagnosis of this rare tumor [2, 7]. Upon dissection, gross appearance of bony tissue is seen and on histopathology, there is typical display of mature fatty tissue with diffuse bone trabeculae [6]. In addition, histopathology also allows the identification of histological subtypes in an already known variants of lipoma, such as low-fat and fat free spindle cell lipomas; this highlights the importance for careful microscopic evaluation of this tumor [18]. For the present case too, although the CT scan and FNAC were performed, the definitive diagnosis of this tumor could only be clinched on histopathological examination of the resected specimen. 

Therapeutically, complete surgical excision is the treatment of choice and due to the benign nature of this tumor, the prognosis is favorable. However, a close monitoring and long-term follow-up of such patients is mandatory as the possibility of malignant transformation and recurrence rate of this tumor cannot be commented upon so far due to the scarcity of clinical data available till date.

## Conclusions

Intraoral osteolipoma is an unusual tumor which occasionally involves the hard palate. Given the rarity, it generally remains unrecognized by the treating clinicians, thus creating diagnostic dilemmas. Hence, osteolipoma should be included as one of the important differentials among the hard palate lesions. Preoperatively, a combination of detailed clinical history, physical examination, cytological, and radiological assessment aid in its diagnosis, but it is the histopathology which is confirmative. Nevertheless, more insight is required in future to understand the genesis and behaviour of this tumor especially in those which occur at atypical sites.
